# A mechanism underlying the effects of polyunsaturated fatty acids on breast cancer

**DOI:** 10.3892/ijmm.2012.1022

**Published:** 2012-06-11

**Authors:** HAO ZHANG, LEI ZHOU, WEI SHI, NING SONG, KARU YU, YUCHUN GU

**Affiliations:** 1 Department of General Surgery, Huashan Hospital, Fudan University, Shanghai;; 2 Department of Cardiology, Jiangsu General Hospital, Nanjing;; 3 Institute of Molecular Medicine, Peking University, Beijing;; 4 Guyuanlong Department of General Surgery, Wuxi Second Hospital, Wuxi, Jiangsu, P.R. China

**Keywords:** MCF-7, TRP channel, arachidonic acid, metabolites, proliferation, migration

## Abstract

Breast cancer is the most frequent cancer in women. Evidence suggests that the polyunsaturated fatty acids (PUFAs), eicosapentaenoic acid (EPA), and docosahexaenoic acid (DHA) affect breast cancer proliferation, differentiation and prognosis. However, the mechanism still remains unclear. In this study, the expression of transient receptor potential canonical (TRPC)3 was detected throughout the cell cytoplasm and at the cell surface of MCF-7 cells. Ca^2+^ entry was induced in these cells via activated TRPC3 by either the diacylglycerol analogue (OAG) or by intracellular Ca^2+^ store depletion. TRPC-mediated Ca^2+^ entry was inhibited by PUFAs including arachidonic acid (AA) and linolenic acid (LA) but not saturated fatty acids. Overexpression of the PUFA degradation enzyme, cyclooxygenase 2 (COX2), enhanced capacitative Ca^2+^ entry. In addition, inhibition of COX2 reduced [Ca^2+^]_i_. Nevertheless, inhibition of TRPC reduced the cell cycle S phase and cell migration, implicating a functional role for TRP-mediated Ca^2+^ entry in cell proliferation and invasion. Exogenous PUFA as well as a TRPC3 antagonist consistently attenuated breast cancer cell proliferation and migration, suggesting a mechanism in which PUFA restrains the breast cancer partly via its inhibition of TRPC channels. Additionally, our results also suggest that TRPC3 appears as a new mediator of breast cancer cell migration/invasion and represents a potential target for a new class of anticancer agent.

## Introduction

Breast cancer is the most frequent cancer in women (1). Evidence suggests that polyunsaturated fatty acids (PUFAs), including arachidonic acid (AA) and linolenic acid (LA) but also eicosapentaenoic acid (EPA) and docosahexaenoic acid (DHA), influence breast cancer proliferation (2–4), differentiation (3), and prognosis (5). Clinical and research data in the past 20 years reveal that cyclooxygenase (COX)2 is overexpressed in a variety of malignant tumors (6–8) and are linked to apoptosis resistance (9), invasive tumor behavior (10) and the poor prognosis of breast cancer (8,11,12). However, the mechanism of unsaturated fatty acids upon breast cancer still remains unclear.

Ca^2+^ is one of most important signal transduction elements in cells ranging from bacteria to neurons. The molecular identity of the membrane protein that serves to enable capacitative Ca^2+^ entry (CCE) following the functional depletion of intracellular Ca^2+^ store or activation of G-protein (13) has yet to be determined with any certainty, but the canonical, vertebrate TRPs (TRPC) are widely believed to fulfil this vital role in many cell types (14,15). Cell-specific, differential expression of TRPC has been described in many excitable and non-excitable cell types, including prostate cancer cells (16) and breast cancer cells (17,18) where they contribute and mediate the Ca^2+^ entry in response to multiple physico-chemical stimuli (19,20).

The PUFA, AA has been proposed to activate TRPC, in many mammalian cell types, including endothelial (21,22), epithelial and smooth muscle (23) cells, but the direct evidence for this in these cells is lacking and the proposals are based primarily upon findings in *Drosophila* TRP and TRP-like (TRPL) (24) and mammalian TRPV channels (25) where AA and LA induce activation, leading to Ca^2+^ entry.

COX acts to degrade AA. In addition, high cellular levels of COX are commonly used as a marker for malignant breast cancer (6,10,12). This suggests that AA and/or its degenerate products may play a role in this pathological process.

In this study, we found the functional expression of TRPC3 in human MCF-7 breast cancer cell-mediated Ca^2+^ entry. Native TRPC channels in MCF-7 cells were inhibited by PUFA. Ca^2+^ entry via activated TRPC was enhanced when PUFA were absent, suggesting a double-gating mechanism for TRPC that may be involved in MCF breast cancer cell proliferation and invasion.

## Materials and methods

### Cell culture

MCF-7 cells were grown in DMEM medium containing 10% fetal calf serum and 1% penicillin/streptomycin serum as described (9). Cells were plated onto ø13-mm coverslips and used when 60–70% confluent.

### Calcium imaging

The growth medium was removed and cells were rinsed once in Earle’s balanced salts solution (EBSS; Invitrogen). Calcium-green of 50 μg AM (C3012; Invitrogen) or Fura-2 AM (F1221; Invitrogen) were dissolved in 20 μl 20% pluronic acid in DMSO (0.01 g in 50 μl DMSO stock). Before the experiment, mixtures of 1 μl dye preparation in 200 μl EBSS was applied and cells were incubated for 60 min. Prior to placing the coverslip into the recording chamber, coverslips were rinsed in EBSS to remove residual dye. Data acquisition and analysis were performed via OpenLab v.3.1.7 (Improvision Ltd., Coventry, UK). A CCD camera (ORCA-AG; Hamamatsu Ltd., Japan) was used to capture the fluorescent image by using Fura-2-AM and calcium green. In the experiments performed using Fura-2, fluorescent intensities were measured with dual-sequential-wavelength excitation at 340 and 380 nm, and emission at 510 nm. Changes in Ca^2+^ concentration were expressed as ratios of 340/380. Fluorescent intensity of calcium green-1 was measured with a single wavelength excitation at 488 nm and emission at 528 nm. Changes in the Ca^2+^ concentration were expressed as ΔF/F, where F was the fluorescence intensity when cells were at rest, and ΔF was the change in fluorescence during stimulation.

### iRNA and plasmid of hCOX2

Stealth siRNA (Invitrogen) was obtained from Invitrogen. MCF-7 cells were passaged onto coverslips in 500 μl Opti-MEM (Invitrogen) one day before transfection and reached about 40–50% confluence at the time of transfection. siRNA of 20 pmol (against TRPC3) or the siRNA negative control complex, with a 1:125 final dilution of Lipofectamine 2000 (Invitrogen) was used according to the manufacturer’s instructions. The knockdown effects were examined at 48 h and the results were compared with control and control without knockdown. Results were collected from 3 different batches of MCF-7 cells. Human hCOX2 plasmids were obtained from Professor R. Kulmacz (University of Texas Health Science Center at Houston). Cells were transfected with hCOX2 by Lipofectamine 2000. The effects of transfection were examined by western blot analysis at 24 and 48 h.

### RT-PCR and immunostaining

RT-PCR experiments followed standard protocols. Primers were designed with primer 3 software (http://frodo.wi.mit.edu/cgi-bin/primer3/primer3_www.cgi) for TRPC1 (NM_003304/92 bp), TRPC3 (NM_003305/157 bp), TRPC4 (NM_016179/191 bp), TRPC5 (NM_012471/108 bp), TRPC7 (NM_020389/135 bp) and the α1C subunit (NM_000719/194 bp), α1G subunit (AH_007322/135 bp) and α1H subunit of VGCCs (NM_021098/123 bp). Antibodies against TRPC1, 3, 4 and 5 were the kind gift from Professor W.P. Schilling (Case Western University, Cleveland, OH, USA). The peptide sequence (26) used to generate the antibody against TRPC3 was RRRRLQKDIEMGMGN.

### Cell cycle analysis

After removal of methanol, cells were treated with a Coulter DNA-Prep reagent kit (Beckman-Coulter, France). Cells were resuspended in 40 μl of a lysing and permeabilizing reagent and 400 μl of a propidium iodide solution containing RNAse. Flow cytometry analyses were performed using a Coulter Epics Elite ESP flow cytometer (Beckman-Coulter) equipped with a 488 nm argon laser running at 15 mW. The red DNA fluorescence signal was analyzed as total (area) vs. peak signal, in order to eliminate doublets and aggregates. Data were recorded for at least 10,000 events. Cell cycle distribution was analyzed with the Multicycle-AV software (Phoenix Flow Systems, San Diego, CA, USA).

### Cell survival and proliferation

Cell proliferation was determined using the tetrazolium salt reduction method (MTT). Cells were seeded at 4×10^4^ cells/well on a 24-well plate (for a given condition on three separate experiments) and allowed to start growth for 48 h. Drugs and AA were added for 24 and 48 h at the concentrations indicated in the figure legends. Cells were incubated at 37°C with the tetrazolium salt [3-(4,5-dimethylthiazol-2-yl)-2,5-diphenyl tetrazolium bromide] and metabolically active cells reduced the dye to purple formazan. After 45 min of incubation at 37°C, the medium was discarded and MTT formazan crystals were solubilized with DMSO. Absorbance was measured in a multiwell plate spectrophotometer at 570 nm (Molecular Devices model Thermomax microplate reader, Les Ulis, France). For easier comparison between conditions, results obtained for proliferation were normalized. The means were then calculated on the daily calculated ratios. The mean of each triplicate was used to create a data point for comparing cell growth in different conditions.

### Cell migration/invasion in vitro

Invasion and migration were analyzed in BD Falcon 24-well plates with 8-μm pore size polyethylene terephtalate membrane BD BioCoat cell culture inserts. For the invasion assay, the membrane was covered with a film of Matrigel (Becton-Dickinson). The upper compartment was seeded with 4×10^4^ viable cells in DMEM with 5% FBS ± drugs/AA and the lower compartment was filled with DMEM supplemented with 10% FBS as a chemo-attractant ± drugs/AA. After 48 h at 37°C, cells remaining on the upper side on the membrane were removed with a cotton swab and the cells that had migrated and were attached to the lower side were stained with hematoxylin for 2 min and counted on the total surface of the insert using light microscopy at x200 magnification.

### Solution and chemicals

EBSS for calcium imaging recording contained: NaCl 116.3 mM, NaH_2_PO_4_ 1 mM, KCl 5.3 mM, MgCl_2_ 1 mM, CaCl_2_ 1.8 mM, NaHCO_3_ 26 mM, D-glucose 5.5 mM, HEPES 10 mM. The EGTA solution for calcium imaging recording contained: NaCl 116.3 mM, NaH_2_PO_4_ 1 mM, KCl 5.3 and 1.8 mM, NaHCO_3_ 26 mM, D-glucose 5.5 mM, HEPES 10 mM, EGTA 0.2 mM. All solutions were prepared fresh on the day of experimentation.

Chemicals dissolved in ethanol or DMSO were made up as 1,000 times stock solutions. All 1X chemical solutions were made with an appropriate bath solution on the day of experimentation. Solutions such as TG, AA, ETYA, were kept in the dark and on ice and added into a syringe which led to the recording chamber when necessary. The solvent, ethanol at the same dilution, was tested alone in controls and had no effects on calcium entry evoked either by store depletion or OAG stimulation.

## Results

### TRPC3 and the α1H subunit of the voltage gated calcium channel (VGCC) expressed in MCF-7 breast cancer cells

**Figure 1.**
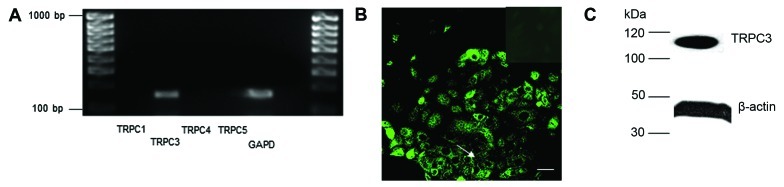
Expression of TRPC3 in MCF-7 cells. (A) The expression of TRPC channels in MCF-7 cells was examined by RT-PCR. The PCR reaction yielded RNA transcript, of expected size, for TRPC3 but not TRPC1, 4 or 5. GAPD positive controls were found in each sample. (B) Immunostaining of TRPC3 in MCF-7 breast cancer cells. TRPC3 expression was seen throughout the cell cytoplasm and cell membrane. Arrows indicate location of antibody in cell membrane. Inset at the top right corner shows peptide negative control (26). Scale bar, 40 μm. (C) Western blot analyses showing the presence of TRPC3 protein in MCF-7 breast cancer cells.

Expression of the TRPC3, α1H (T type) but not the α1C or α1G subunits of the voltage-gated calcium channel (VGCC) was observed in MCF-7 cells (Fig. 1).

The spatial distribution of TRPC3 protein in MCF-7 cells was determined by immunohistochemistry, as previously reported in other cell types (27). Abundant, non-discrete TRPC3 expression throughout the cell cytoplasm and at the cell surface was observed (Fig. 1B). Western blot analysis consistently demonstrated abundant expression of TRPC3 in MCF-7 cells (Fig. 1C).

### Native TRPC3 in MCF-7 breast cancer cells activated by DAG and store depletion

**Figure 2.**
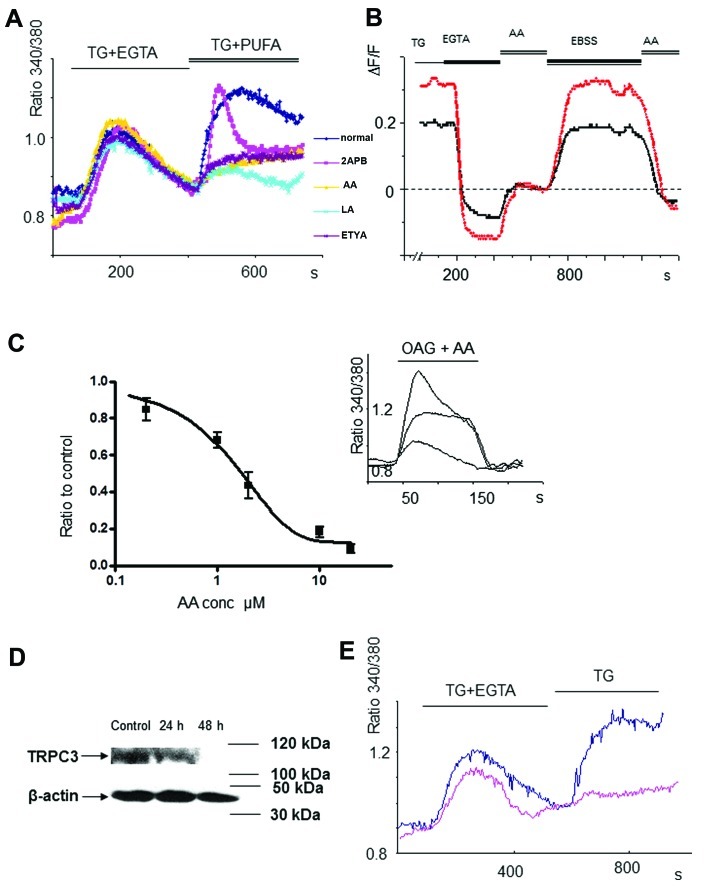
Polyunsaturated fatty acids inhibited Ca^2+^ entry through TRPC3 channels. (A) Ca^2+^ entry was evoked by store depletion with thapsigargin (TG, 1 μM) and EGTA (blue line). 2-APB (pink line, 100 μM) induced a transient Ca^2+^ elevation following a rapid decrease. [Ca^2+^]_i_ elevation was inhibited by arachidonic acid (AA, yellow line, 10 μM), linonic acid (LA, 10 μM) and ETYA (brown line, 20 μM). (B) The dashed horizontal line indicates the basal Ca^2+^ level. TG (1 μM) elevated cellular Ca^2+^ levels that were rapidly reduced to below basal levels by wash out with EGTA (200 μM). Application of arachidonic acid (AA, 10 μM) prevented the overshooting response seen in (A) and only returned Ca^2+^ to the basal level, indicating a block of the TRPC-mediated Ca^2+^ entry pathway by AA. Wash off of AA with EBSS caused an overshooting response to occur that could be blocked, as previously, by application of AA (10 μM). (C) Log dose response curve of varying concentrations of AA upon the OAG-induced Ca^2+^ elevation. Responses were quantified as the area under the Ca^2+^ response curve and normalized by determining the area in the presence of AA, expressed as a fraction of the control response. Curve fit was with a single sigmoidal function. The inlet shows an example of different concentration-dependent effects of AA upon Ca^2+^ entry elicited by OAG (50 μM). (D) Western blot analysis demonstrates that protein levels of TRPC3 channels gradually diminished after 48 h. (E) Ca^2+^ entry evoked by store depletion was significantly reduced 48 h after incubation of MCF-7 cells with RNAi against TRPC3, . Results were averaged from 3 coverslips except where indicated.

Recombinant human TRPC3 can be directly activated by diacylglycerol (DAG) (28,29). In MCF-7 cells, OAG (100 μM), a synthetic DAG, induced a significant intracellular Ca^2+^ elevation in an extracellular calcium-dependent manner (Fig. 2). Recent reports have confirmed that TRPC3 could also mediate store operated calcium entry (SOCs) (30). Activation of native TRPC3 via store depletion by SERCA pump inhibitors, such as TG, has been reported in HSG (31) and epithelial cells (32). In MCF-7 cells, store depletion by TG (1 μM) and EGTA bath led to significant Ca^2+^ elevations (Fig. 2A).

### AA and LA directly inhibited calcium entry via native TRPC3

The Ca^2+^ elevation induced by store depletion was almost quenched by bath application of 10 μM AA (Fig. 2A, yellow line) or 10 μM LA (Fig. 2A, blue line). 2APB, as a common antagonist of TRPC3, at the concentration of 100 μM, caused a transient calcium peak followed by decay (Fig. 2A, pink line) to a level similar to that in the presence of AA or LA. In addition, the CCE following Ca^2+^ replacement after EGTA, could be prevented by 10 μM AA and CCE was observed after removal, by washout, of the AA inhibition (Fig. 2B).

### Effect of PUFA on TRPC3 not via the degenerated products of PUFA

Exogenous AA can freely penetrate membranes, but will be challenged and degraded by endogenous intracellular cyclooxygenases (COX) or lipooxygenases (LOX). To exclude the possibility that the inhibition effect is due to degenerated metabolites of AA rather than AA itself, 14-eicosatetraynoic acid (ETYA, 20 μM), a competitive analogue of AA resistant to LOX and other degradative enzymes, was used to mimic the effect of AA. ETYA, like AA, inhibited CCE induced by store depletion (Fig. 2A, brown line), suggesting that PUFA interacts directly with TRPC channels.

The effect of fatty acids on ion channels is sometimes attributed to non-specific influences upon cell membranes (33,34). Polyunsaturated fatty acids may, for example, increase membrane fluidity. AA at a concentration between 25–100 μM directly affected channel proteins via a change of the lipid environment (34). We observed that application of 500 μM AA induced a massive, irreversible Ca^2+^ elevation, presumably following the collapse of cell integrity. We therefore maintained PUFA concentrations below the threshold (15 μM) for such effects. Additionally, CCE was also inhibited by the double-bonds unsaturated fatty acid, e.g. LA (5 and 10 μM) and the mono-bond unsaturated fatty acid, oleic acid (10 μM). However, the saturated fatty acid, stearic acid (20 μM), had no effect upon induced CCE.

To confirm that CCE in MCF-7 cells was mediated via TRPC3 channels, we examined Ca^2+^ entry in MCF-7 cells by knocking down TRPC3 (shealth RNAi; Invitrogen) (Fig. 2D and E). A negative control iRNA was utilized as a control to discount the non-specific effect of TRPC3 iRNA. Transfected cells gradually lost the TRPC3 channels and after 48 h, detectable TRPC3 protein was almost eliminated (Fig. 2D). In parallel to the decrease of TRPC3 protein, calcium elevation evoked by store depletion was also significantly reduced; suggesting that calcium elevation induced by store depletion was mediated via the TRPC3 channels.

### Dose response of AA inhibition upon calcium entry via TRPC3

Ca^2+^ entry in MCF-7 cells could be evoked by application of OAG (Fig. 2C). AA in a range from 0.1 to 20 μM was employed to study the inhibitory effect of AA on OAG induced Ca^2+^ elevation. A single, sigmoidal dose-response curve was fitted according to data (Fig. 2C) and gave an IC_50_ of AA upon OAG induced Ca^2+^ elevation of 1.78±0.17 μM.

### Regulation of endogenous AA in MCF-7 and its effect on Ca^2+^ entry via TRPC3

There are generally two principal sources in AA generation, i) cytosolic phospholipase A2 (PLA_2_) releasing AA from appropriate phospholipids (35–37) and ii) fatty acid amide hydrolase (FAAH) degenerating endogenous anandamide and relatives into AA. The AA metabolism diverges down two main pathways, the COX and LOX pathways (38).

**Figure 3.**
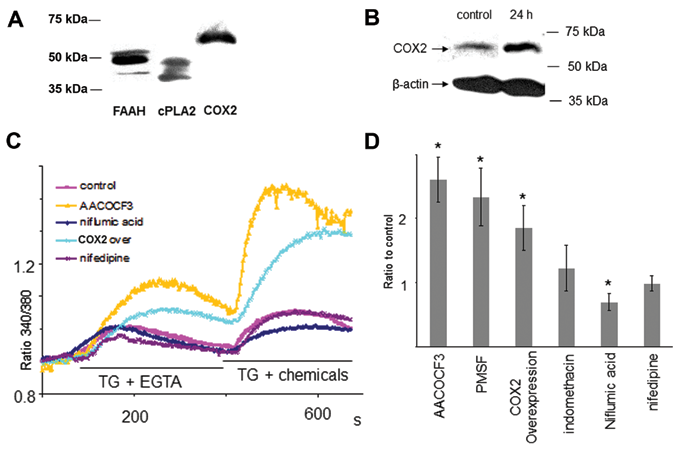
Modulation of endogenous PUFA and its effect on TRPC3 mediated Ca^2+^ entry. (A) Western blot analyses demonstrate the presence of fatty acid hydoxylase (FAAH), cytoplasmic phospholipase A (cPLA) and COX2 in MCF-7 breast cancer cells. (B) COX2 protein levels were dramatically increased (almost 3-fold) 24 h after cells were transfected with COX2 plasmid. (C) Ca^2+^ entry evoked by store depletion was enhanced from control (pink line) by reduction of AA generation e.g. AACOCF3 (yellow line, 10 μM), COX2 expression (blue line). Nifedipine (purple line, 20 μM) did not affect Ca^2+^ entry but niflumic acid (blue line, 100 μM) as a COX2 inhibitor, slightly reduced the Ca^2+^ entry induced by store depletion. (D) All results were normalized to the controls. AACOCF3, PMSF (50 μM), and overexpression of COX2 significantly enhanced Ca^2+^ entry via activated TRPC induced by store depletion. Meanwhile niflumic acid reduced Ca^2+^ entry via TRPC induced by store depletion. Indomethacin and nifedipine did not affect Ca^2+^ entry via TRPC3.

cPLA2, FAAH and COX2 were demonstrated to be present in MCF-7 breast cancer cells by western blot analyses (Fig. 3A). Accordingly, reduction of endogenous PUFA could be achieved by either inhibition of cPLA2, FAAH or overexpression of COX2. Inhibition COX2 or LOX could induce the elevation of endogenous PUFA. Consistent with our results above, reduction of endogenous PUFA by the cPLA2 antagonist (AACOCF3), FAAH antagonist (PMSF), and overexpression of COX2 significantly enhanced Ca^2+^ entry via TRPC3. Niflumic acid, the antagonist of COX2, reduces degeneration of PUFA, resulting in elevation of endogenous PUFA. Ca^2+^ entry via TRPC3 was reduced by pre-incubation with niflumic acid (Fig. 3D).

### Effect of TRPC antagonism on MCF-7 cell proliferation and invasion

**Figure 4.**
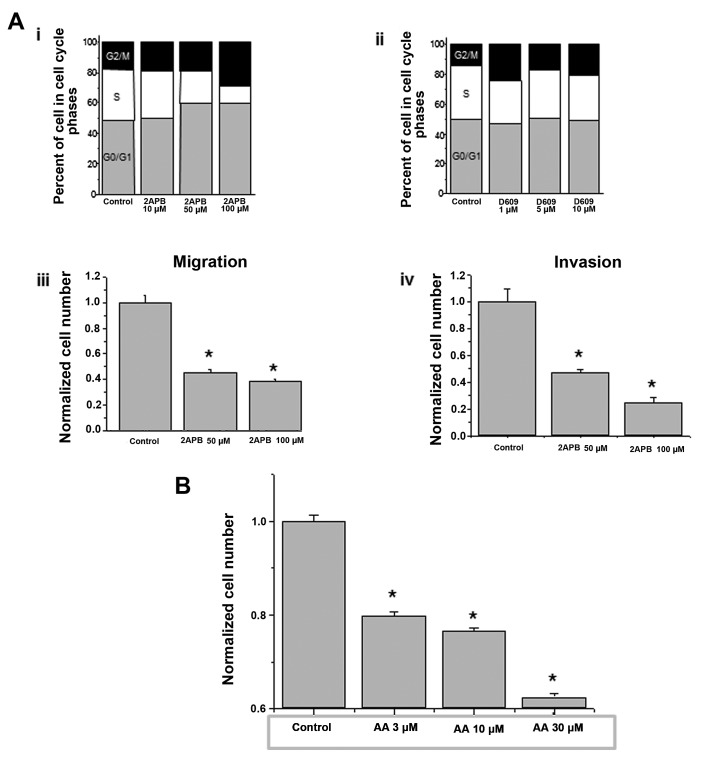
(A) Antagonists of TRPC and AA interfer with the cell cycle and the migration of MCF-7 breast cancer cells. (i) 2-APB caused a dose-dependent and selective decrease in the S phase of the MCF-7 cell cycle compared to the vehicle control. (ii) D609 had no effect on the cell cycle parameters measured. (iii) 2-APB caused a significant decrease in the migration of MCF-7 cells. (iv) 2-APB caused a dose-dependent and significant decrease in the invasion of MCF-7 cells. (B) The normalized bar figure shows the effects of different concentrations of AA on cell migration.

The common antagonist of TRP channels, 2-APB, was used to investigate the effects of these channels on the proliferation of MCF-7 breast cancer cells. 2-APB (50 μM) significantly reduced the S phase of the cell cycle (Fig. 4A-i), and cell proliferation (MTT test; 100 μM), whilst D609, an inhibitor of phospholipase C, had no effect (Fig. 4A-ii). 2-APB also reduced MCF-7 cell migration and invasion in a dose-dependent manner (Fig. 4A-iii and iv). The effects of increasing concentration of AA on cell migration are shown in Fig. 4B. Taken together, these results suggest that cellular AA inhibits Ca^2+^ entry via TRPC3, thus interfering with the Ca^2+^ requirement for cell proliferation and invasion.

## Discussion

In this study, we found that TRPC3 channels were highly expressed in MCF-7 breast cancer cells. Not only DAG but also store depletion activated TRPC3 to mediate Ca^2+^ entry. [Ca^2+^]_i_ was thereafter crucial to determine breast cancer cell proliferation and migration. PUFAs, such as AA and LA, but not saturated fatty acids, inhibited Ca^2+^ entry mediated by TRPC3. Channel activation and removal of PUFAs were required to allow Ca^2+^ entry via TRPC. Endogenous local PUFAs regulated by cellular generation and degeneration pathways therefore played an important role in mediating [Ca^2+^]_i_ via TRPC. PUFAs as well as the TRPC3 antagonist attenuated breast cancer cell proliferation and migration, suggesting a mechanism in which PUFAs restrain breast cancer via inhibition of TRPC channels.

TRP channels play a key role in the regulation of intracellular Ca^2+^ in multiple cell types e.g. sperm (39), smooth muscle (40) and prostate cancer cells (41) in response to multiple stimuli. At least four of the TRPC family members (TRPC1, 2, 4 and 5) are known to be activated by store depletion, while TRPC3 and 6 are generally regarded to be activated by DAG (28,29). However, native TRPC3 in different cells e.g. avian pre-B cells (42), ROS 17/2.8 (43) and PS1 prostate cancer cells (44) are also reported to be activated by store depletion. The proportion of activation in these cells, induced by DAG or by store depletion, may vary with different levels of TRPC3 expression. It has been reported that expression levels of the TRPC3 channel might determine the actual activation mechanism (45). Inconsistent to previous studies, we found that TRPC3 in MCF-7 breast cancer cells are activated by DAG as well as by store depletion. The expression of TRP in MCF-7 cells may vary (18) due to the culture methods. In hBDC, TRPC1, TRPC6, TRPM7, TRPM8 and TRPV6 channels were overexpressed in hBDA compared to the adjacent non-tumoral tissue. TRPC1, TRPM7 and TRPM8 expression strongly correlated with proliferative parameters, and TRPV6 was mainly overexpressed in invasive breast cancer cells (18). However, in the MCF-7 cell line in our laboratory, TRPC1 was not expressed.

PUFAs have been found to directly activate *Drosophila* TRP and TRPL (24) as well as TRPV in vascular endothelial cells (25). However, there is little evidence to show that PUFA directly activates native TRPC in mammalian tissues rather than in expression systems. Interestingly, Ca^2+^ entry mediated by AA has been reported (46–48), but it is ascribed to a novel receptor-activated calcium entry pathway, ARC (49,50), as there appeared to be mutual antagonism of CCE and ARC (51–53). In our experiment, the application of PUFA alone did not cause calcium elevation, and selective TRPC3 iRNA inhibited the calcium elevation in response to store depletion. We therefore ruled out the possibility of calcium entry via ARC. Other studies on the effect of AA on voltage-gated Kv channels suggest that AA closed Kv channels by introducing conformational alternations in selective filter regions of the channel (54). How PUFAs interact with the TRPC3 protein within internal membranes, at present, is still unclear.

The lack of specific antagonists for TRP channels creates difficulties in determining the molecular basis of TRP channels. 2-APB has a broad effect on TRPC, IP3 and TRPV. The silent response of MCF-7 cells to CAP, the most potent TRPV agonist, ruled out the possible involvement of TRPV in the mediation of calcium entry in MCF-7 cells. Consistently, western blot analyses against TRPV1 in MCF-7 cells failed to detect the expected protein, compared to positive controls from rat brain. In addition, the potent inhibition of RNAi against TRPC3 on the Ca^2+^ entry induced by store depletion suggests that the inhibition of AA on Ca^2+^ entry occurred via its effect on TRPC3.

The concentration of free AA in resting cells is universally described as low but varies from cell to cell, AA concentrations in the resting leukocyte have been measures at 0.5–1 μM as opposed to at 15 μM in the islets of Langerhans (55). This resting concentration can be varied dynamically by either increases, for example via activation of G-protein coupled receptors and phospholipase A2 (56), or decreases via AA degenerative pathways mediated principally by COX and LOX. As there are multiple buffer systems for AA, the free intracellular AA concentrations correlating with the bath AA concentration are not easily determined.

As expected, reduction of endogenous AA by inhibition of cPLA2, FAAH and overexpression of COX2 increase Ca^2+^ entry evoked by store depletion. However, the effect of increasing endogenous AA by inhibition of COX2 and/or LOX was not of significance, suggesting some unknown mechanism. In addition, inhibition of FAAH was complicated, because AEA alone causes calcium elevations (data not shown).

In addition, COX2 has been shown to be overexpressed in a variety of malignant tumors (6,7) and linked to apoptosis resistance (9), invasive tumor behavior (10) and the poor prognosis of breast cancer (12). Our findings suggest a mechanism whereby AA, through direct inhibition of Ca^2+^ entry via TRPC3 channels may play a role in cancer cell proliferation and invasion.

In conclusion, we have demonstrated that polyunsaturated fatty acids directly inhibit TRPC3 in MCF-7 cells and is a potent mechanism for regulating Ca^2+^ entry and [Ca^2+^]_i_. Our data suggests a role for such regulation in breast cancer metastasis. Thus, TRPC3 appears as a new mediator of breast cancer cell migration/invasion and represents a potential target for a new class of anticancer agents.

## Abbreviations:

TRP: transient receptor potential;
PUFA: polyunsaturated fatty acids;
CCE: capacitative Ca^2+^ entry;
VGCC: voltage-gated calcium channel;
SER: smooth endoplasmic reticulum;
TG: tharpsigargin;
AA: arachidonic acid;
LA: linoleic acid;
COX: cyclooxygenases;
LOX: lipooxygenases;
ETYA: 14-eicosatetraynoic acid;
EPA: eicosapentaenoic acid;
DHA: docosahexaenoic acid.
